# Deregulated expression of cryptochrome genes in human colorectal cancer

**DOI:** 10.1186/s12943-016-0492-8

**Published:** 2016-01-15

**Authors:** Gianluigi Mazzoccoli, Tommaso Colangelo, Anna Panza, Rosa Rubino, Angelo De Cata, Cristiana Tiberio, Maria Rosa Valvano, Valerio Pazienza, Giuseppe Merla, Bartolomeo Augello, Domenico Trombetta, Clelia Tiziana Storlazzi, Gemma Macchia, Annamaria Gentile, Francesca Tavano, Manlio Vinciguerra, Giovanni Bisceglia, Valeria Rosato, Vittorio Colantuoni, Lina Sabatino, Ada Piepoli

**Affiliations:** Division of Internal Medicine and Chronobiology Unit, IRCCS Scientific Institute and Regional General Hospital “Casa Sollievo della Sofferenza”, San Giovanni Rotondo, FG Italy; Department of Sciences and Technologies, University of Sannio, Benevento, Italy; Division of Gastroenterology and Research Laboratory, IRCCS Scientific Institute and Regional General Hospital “Casa Sollievo della Sofferenza”, San Giovanni Rotondo, FG Italy; Medical Genetics, IRCCS Scientific Institute and Regional General Hospital “Casa Sollievo della Sofferenza”, San Giovanni Rotondo, FG Italy; Oncology-Research Laboratory, IRCCS Scientific Institute and Regional General Hospital “Casa Sollievo della Sofferenza”, San Giovanni Rotondo, FG Italy; Department of Biology, University of Bari, Bari, Italy; Euro-Mediterranean Institute of Sciences and Technology (IEMEST), Palermo, Italy; School of Science and Technology, Nottingham Trent University, Nottingham, UK; Division of Medicine, University College London, Institute for Liver and Digestive Health, Royal Free Campus, London, UK; Division of Abdominal Surgery, IRCCS Scientific Institute and Regional General Hospital “Casa Sollievo della Sofferenza”, San Giovanni Rotondo, FG Italy; Division of Epidemiology and Health Statistics, IRCCS Scientific Institute and Regional General Hospital “Casa Sollievo della Sofferenza”, San Giovanni Rotondo, FG Italy; Department of Medical Sciences, Division of Internal Medicine and Chronobiology Unit, IRCCS Scientific Institute and Regional General Hospital “Casa Sollievo della Sofferenza”, San Giovanni Rotondo, FG 71013 Italy

**Keywords:** Clock gene, Cryptochrome, p53, Colorectal cancer, Chronotherapy, Circadian

## Abstract

**Background:**

Circadian disruption and deranged molecular clockworks are involved in carcinogenesis. The cryptochrome genes (*CRY1* and *CRY2*) encode circadian proteins important for the functioning of biological oscillators. Their expression in human colorectal cancer (CRC) and in colon cancer cell lines has not been evaluated so far.

**Methods:**

We investigated *CRY*1 and *CRY*2 expression in fifty CRCs and in the CaCo2, HCT116, HT29, SW480 cell lines.

**Results:**

*CRY*1 (*p* = 0.01) and *CRY*2 (*p* < 0.0001) expression was significantly changed in tumour tissue, as confirmed in a large independent CRC dataset. In addition, lower *CRY*1 mRNA levels were observed in patients in the age range of 62-74 years (*p* = 0.018), in female patients (*p* = 0.003) and in cancers located at the transverse colon (*p* = 0.008). Lower *CRY*2 levels were also associated with cancer location at the transverse colon (*p* = 0.007). CRC patients displaying *CRY*1 (*p* = 0.042) and *CRY*2 (*p* = 0.043) expression levels over the median were hallmarked by a poorer survival rate. Survey of selected colon cancer cell lines evidenced variable levels of cryptochrome genes expression and time-dependent changes in their mRNA levels. Moreover, they showed reduced apoptosis, increased proliferation and different response to 5-fluorouracil and oxaliplatin upon *CRY*1 and *CRY*2 ectopic expression. The relationship with p53 status came out as an additional layer of regulation: higher CRY1 and CRY2 protein levels coincided with a wild type p53 as in HCT116 cells and this condition only marginally affected the apoptotic and cell proliferation characteristics of the cells upon *CRY* ectopic expression. Conversely, lower CRY and CRY2 levels as in HT29 and SW480 cells coincided with a mutated p53 and a more robust apoptosis and proliferation upon *CRY* transfection. Besides, an heterogeneous pattern of *ARNTL*, *WEE* and *c-MY*C expression hallmarked the chosen colon cancer cell lines and likely influenced their phenotypic changes.

**Conclusion:**

Cryptochrome gene expression is altered in CRC, particularly in elderly subjects, female patients and cancers located at the transverse colon, affecting overall survival. Altered *CRY*1 and *CRY*2 expression patterns and the interplay with the genetic landscape in colon cancer cells may underlie phenotypic divergence that could influence disease behavior as well as CRC patients survival and response to chemotherapy.

**Electronic supplementary material:**

The online version of this article (doi:10.1186/s12943-016-0492-8) contains supplementary material, which is available to authorized users.

## Background

Biological phenomena underling physiology and behavior show rhythms with about 24-h (circadian) periodicity featuring a wide range of cellular and tissue processes as well as organ functions. Nycthemeral rhythmicity is driven by the circadian timing system, a hierarchical structure hard-wired by central and peripheral biological oscillators [1, 2]. The central oscillators are located in the suprachiasmatic nuclei (SCN) of the anterior hypothalamus, small regions positioned at each side of the III ventricle and composed of approximately 20.000 neurons in mice and 100.000 neurons in humans [1, 2]. The SCN respond to external cues, principally photic information transmitted through the retino-hypothalamic tract and dictated by the environmental light-darkness alternation, and drive peripheral oscillators present in nearly all body tissues and organs via neural pathways (autonomic nervous system) as well as humoral outputs (melatonin, cortisol) [1, 2]. The biological clocks endowed in central and peripheral oscillators are operated by molecular clockworks ticking by means of a transcription-translation feedback loop encompassing a set of circadian genes and proteins and revolving with a free-running period of about 24 h [3, 4]. The negative limb of this loop relies on period (*PER* 1-3) and cryptochrome (*CRY*1 and *CRY*2) genes activated by heterodimers of the transcription factors CLOCK and ARNTL (also called BMAL1). PER and CRY proteins form a repression complex in the cytoplasm, which translocates into the nucleus and interacts directly with CLOCK and ARNTL [5]. The casein kinase (CK) Iδ and CKIε may target them for degradation and regulate their nuclear translocation [6]. CLOCK and ARNTL also activate the expression of *NR1D1* and *Rora* genes, encoding the nuclear receptors REV-ERBα and RORα, respectively. The rhythmic transcription of *ARNTL* is also regulated by these receptors, as REV-ERBα prevents the binding of the positive transcriptional regulator RORα to specific response elements in gene promoters [7, 8]. The clock gene machinery controls cell metabolism, proliferation, differentiation, DNA damage response, apoptosis, and autophagy [9]. In particular, the chryptochrome genes influence cell cycle progression and lack of Cry proteins in cultured primary *Cry1*^*-/-*^*Cry2*^*-/-*^ mouse fibroblasts is associated with faster cell proliferation [10]. Disruption of the circadian clock may lead to deregulated cellular processes driving carcinogenesis, in particular in colorectal tissues and may influence the response to chemotherapeutic agents [11–13]. *Cry1*^*-/-*^*Cry2*^*-/-*^ double knockout mice are hallmarked by constitutively high resistance to cyclophosphamide, and show a constant active state of CLOCK:BMAL1 heterodimers, inducing high level expression of target genes at any time of the circadian cycle [14]. Besides, *CLOCK* gene amplification and overexpression was associated with a high risk for colorectal cancer (CRC) and with poor prognosis in CRC patients. Experiments performed in vitro showed that *CLOCK* up-regulation propped up proliferation and restrained apoptosis in SW480 cells, whereas *CLOCK* down-regulation slowed down proliferation and speeded up apoptosis in SW620 cell. At the molecular level, *CLOCK* over-expression induced significant reduction of Bax and Bid expression as well as significant increase in p‑AKT expression, while *CLOCK* silencing induced significant diminution in p‑AKT expression without influencing total AKT levels [15].

CRC is the third most common type of human cancer in both sexes and the second most common cause of cancer death in Western countries [16, 17]. Surgical intervention in early stages represents the only effective treatment, whereas chemotherapy has modest effects, suggesting the need for new prognostic molecular biomarkers and therapeutic approaches.

Advances in molecular chronobiology have led to the development of cancer chronotherapy, which refers to the use of rhythmic cycles in the application of therapy. Following a time qualified treatment schedule, the intent is to promote both the anti-cancer action of drugs, and limit the drug-related side effects [18]. On the premise that circadian clocks control cellular proliferation and drug metabolism over the 24 h, previously untreated CRC patients bearing unresectable liver metastases were treated with chronomodulated chemotherapy regimen with 5-fluorouracil (5FU), leucovorin, and oxaliplatin (OXA), called chronoFLO4 [19]. Unfortunately, this latter approach offered no survival advantage as compared to conventional chemotherapy, and differences in toxicity and outcome between male and female patients were apparent [20]. Indeed, chemotherapeutic agents attenuate the oscillating expression of circadian genes, causing chronodisruption, particularly in female patients, attributable to gender related differences in circadian cycle [21]. The search for reliable and valuable circadian biomarkers for non invasive monitoring is necessary to define the optimal circadian timing of chemotherapy [22].

Sex dimorphism in mice and humans impinges on hepatic drug metabolism [23], and dimorphic liver metabolism is altered when the cryptochrome genes are inactivated. The levels of sex-specific liver products, including several cytochrome P450 enzymes, expressed by *Cry1*^*-/-*^*Cry2*^*-/-*^ male mice are similar to those expressed by female mice. Besides, an altered pattern of circulating growth hormone (GH) has been evidenced in *Cry1*^*-/-*^*Cry2*^*-/-*^ male mice, suggesting a 24-h clock control and pacing on the dimorphic ultradian pulsatility of GH, which is responsible for sex-dependent liver activity [24].

Uncovering the relationships among circadian timing, sex dimorphism and liver metabolism would be crucial to customize chronotherapy. The aim of our study was to evaluate the expression patterns of *CRY*1 and *CRY*2 in neoplastic tissues of male and female CRC patients and in colon cancer cell lines in order to validate preliminary results previously obtained by our group [25] and explore the role of cryptochrome genes in colon cancer cell behaviour and response to chemotherapeutic agents.

## Methods

### Sample size

We had to enroll at least 45 patients in order to achieve a power of 80 % with a type I error of 5 % in detecting a gene expression difference of 0.30 between the CRC tumour tissue and normal mucosa, using a two-sided two-sample *t*-test and analysis of variance (ANOVA) (standard deviation of 0.50. A total number of 30 subjects was required for Pearson’s correlation with a correlation coefficient of 0.50 and a type I error of 5 %.

### Patients

This study was approved by the Ethical Board of our Institute, and all patients gave written informed consent. The experimental protocol conformed to international ethical standards. *CRY*1 and *CRY*2 mRNA and protein levels were evaluated in the tumour tissues and adjacent normal tissues of 50 patients (34 men and 16 women, mean age ± SD 67.2 ± 11.6 years) undergoing surgery for primary colorectal cancer at our Hospital. Patient characteristics are shown in Table 1. All tumoral and normal tissue specimens were collected between 9:00 a.m. and 17:00 p.m. of the same day (8 h), dissected immediately, stained with haematoxylin-eosin and analyzed by the pathologist to determine the tumour cell percentage. Tissue samples having at least 80 % of tumour cell content were frozen in liquid nitrogen until the molecular analysis.

**Table 1:** Clinical and pathological features of colorectal cancer patients

	n = 50	%
Age (years):		
Mean ± SD	67.2 ± 11.6	
Gender:		
Male	34	68
Female	16	32
Tumour location:		
Proximal colon^a^	17	34
Transverse colon	6	12
Distal colon^b^	27	54
Histologic Type		
Non-mucinous adenocarcinoma	45	90
Mucinous adenocarcinoma	5	10
Depth of tumour invasion		
T2	4	8
T3	43	86
T4	3	6
Lymph node involvement		
N0	24	48
N1	14	28
N2	12	24
Metastasis		
Not	48	96
Yes	2	4
TNM STAGE		
I-II	27	54
III-IV	23	46
Grading		
G1-G2	45	90
G3-G4	5	10
Modified Dukes Staging System		
A	4	8
B	20	40
C	24	48
D	2	4
Vascular Invasion		
Not	27	54
Yes	23	46
MSI frequency		
Missing	12	24
High	9	18
Low	10	20
Stable	19	38

^a^Caecum and ascending colon

^b^Descending colon, sigmoid colon and rectum

### RNA extraction from fresh frozen tissue and first-strand cDNA synthesis

Total RNA was extracted with TRIzol reagent (Invitrogen) from about 150–200 mg of freshly frozen tissue specimens. The amount of total RNA was determined by UV spectrophotometry using the Nano Drop Spectrophotometer (Nanodrop Technology), and RNA integrity assessed by the Agilent 2100 Bioanalyzer (Agilent Technologies) after digestion by DNaseI. Next, 1.0 μg of total RNA was reversed transcribed using the High-Capacity cDNA Archive Kit following the manufacturer’s instructions (Applied Biosystems).

### Quantitative real-time reverse transcription-PCR assay

To assess the differential expression of the cryptochrome genes in CRC specimens and matched normal mucosa, quantitative real-time PCR (q-PCR) assay was performed by using *CRY1* (QT00025067), *CRY2* (QT00094920), *ARNTL* (QT00011844), *WEE* (QT00038199) and *c-MYC* (QT00035406) Human QuantiTec Primers Assay (SYBR Green QuantiTect Primers Assay; QIAGEN). All qPCRs were performed in a 25-μl final volume, with three replicates per sample, by using QuantiFast SYBR Green PCR kit (QIAGEN) and run in an ABI PRISM® 7700 Sequence Detection System (Applied Biosystems). The data were analyzed using the default and variable parameters available in the SDS software package (version 1.9.1; Applied Biosystems). GAPDH housekeeping control gene was used to normalize target gene expression levels and the mRNA amount of each target gene relative to GAPDH was calculated through the comparative Ct method, also called the 2(−ΔΔCt) method. Two biological replicates were each assayed in triplicate and results were expressed as mean ± standard deviation (SD).

### Microsatellite instability

The Microsatellite instability (MSI) analysis was performed using the Bethesda panel of microsatellite (BAT25, BAT26, D5S346, D17S250 and D2S123) evaluated by means of a multiplex-PCR and PAGE analysis. Tumours showing instability in four or more markers were classified as high MSI (MSI-H), those showing instability in two markers as low MSI (MSI-L), and those showing no instability as microsatellite-stable (MSS).

### Cell culture

CaCo2, HCT116, HT 29 and SW480 colon cancer cell lines were acquired from ATCC (American Type Cell Culture) and cultured as appropriate at 37 °C in 5 % CO2 atmosphere in Dulbecco’s modified Eagle’s medium (DMEM) and Minimum Essential Medium (MEM) Alpha media, supplemented with 10 % fetal bovine serum (FBS), 100 U/ml penicillin and 100 ng/ml streptomycin (Invitrogen Life Technologies). For experiments utilizing synchronized cells and not synchronized cells, CaCo2, HCT116, HT 29 and SW480 cells were seeded at 5 × 10^5^cells/well in 6-well plates. For synchronized cells serum shock was used as synchronization procedure [26]. Briefly, the day of the experiments culture medium was exchanged with serum-rich medium with 50 % FBS only for synchronized cells and after 2 h this medium was replaced with 10 % FBS. The cells were harvested every 3 h over 60 h for *CRY1* and *CRY2* evaluation and every 4 h over 28 h for *ARNTL*, *c-MYC* and *WEE* evaluation. Two biological replicates were each assayed in triplicate and results were expressed as mean ± standard deviation (SD).

### Immunoblot analysis

Tissues and cells were lysed in RIPA buffer (150 mM NaCl, 50 mM Tris-HCl, pH 8, 1 % NP40, 0.1 % SDS, 0.5 % sodium deoxycholate) supplemented with Protease Inhibitor Cocktail Tablets (ROCHE). After centrifugation at 13.000 rpm for 10 min at 4 °C, the supernatants were collected as whole cell protein samples. After boiling at 95 °C for 5 min, an equal amount of proteins was loaded on 10 % polyacrylamide gels and separated by electrophoresis. Protein transfer was performed on PVDF membrane (VWR). Membranes were blocked with 5 % nonfat milk in wash buffer (0.2 M Tris-HCl, pH 7.6, 1.5 M NaCl, 0.1 % Tween 20) and incubated with the specific primary antibodies diluted in blocking solution, at the appropriate dilutions. Following three washes, membranes were incubated at room temperature for 1 h with a secondary anti-rabbit or anti-mouse antibody diluted in blocking solution. After three further washes, proteins were revealed by chemiluminescence (ECL, Amersham Biosciences AB) and the signal detected on an X-ray film (Amersham Biosciences AB). We used the following antibodies: affinity purified rabbit anti-CRY1 antibody (Bethyl Laboratories, INC) diluted 1:1000; affinity purified rabbit anti-CRY2 antibody (Bethyl Laboratories, INC) diluted 1:1000; anti-p53-DO7 antibody (Novacastra) diluted 1:500; anti-β-Actin (C4) from Santa Cruz (DBA) diluted 1:5000; rabbit anti-FLAG antibody (Sigma-Aldrich) diluted 1 μg/ml; anti-β-Actin (C4) from Santa Cruz (DBA) diluted 1:5000; anti-rabbit or anti-mouse IgG, HRP-linked secondary antibody from Cell Signaling (Euroclone) diluted 1:2000. Each western blot analysis was performed five times.

### Fluorescent in situ hybridization

The colon cancer cell lines used in the study were tested by Fluorescent In Situ Hybridization (FISH) to detect copy number alterations of the cytobands harboring the *CRY1* (12q23.3) and C*RY2* genes (11p11.2). BAC (Bacterial Artificial Chromosomes) clones encompassing the two genes were selected according to the February 2009 release (GRCh37/hg19) of the University of California at Santa Cruz (UCSC) Human Genome Browser (http://genome.ucsc.edu). We used RP11-632 M14 (chr12:107,260,199-107,462,448) and RP11-1084E2 (chr11:45,808,132-45,997,341) for *CRY1* and *CRY2*, respectively (Fig. 4). Metaphase spreads were prepared as described elsewhere [27]. FISH experiments and digital images acquisition were carried out as previously described [28].

### Cell transfection

CaCo2, HCT116, HT-29 and SW480 cell lines at 70-80 % confluence were transiently transfected with PFMH-hCry1 or pSO2002 plasmids carrying the full length cDNA for *CRY1* and *CRY2*, respectively, fused in frame at their 5’ end to a DNA segment coding for the FLAG epitope (Plasmid #25843, and Plasmid #25842, respectively; Addgene) or with the empty control plasmid pcDNA™4/myc-His (Plasmid # V863-20; Invitrogen) by using Lipofectamine 2000 (Invitrogen) according to the manufacturer’s instructions. After 6 h of incubation at 37 °C, the transfection medium was replaced with complete medium containing 10 % FBS and the experiments were conducted 72 h later.

### Cell proliferation assay with 3-(4,5-dimethylthiazol-2-yl)-2,5-diphenyltetrazolium bromide (MTT) and evaluation of response to chemotherapeutic agents upon CRY1 and CRY2 ectopic transfection

The cells were seeded onto a 96-well plates in 200 μL medium, at a density of 4 × 10^4^ cells/well, and transfected with *CRY1* or *CRY2* expression vectors. At different time points (24, 48, 72 and 96 h) the MTT solution (5 mg/mL, 20 μL) was added and the plates were incubated for 4 h. The medium was removed and Isopropyl alcohol (200 μL) added to dissolve the formazan crystals. The reduction in cell viability was determined colorimetrically by using a spectrophotometer at λ = 570 nm and 630 nm. The results are expressed as the mean of optical density (OD) from triplicate samples. Three independent experiments were carried out and the results expressed based on the following formula: cell viability (%) = number of cells in transfected group/ number of cells in control group x 100.

### CAsy cell counter

Cell number and density of viable cells were determined using CAsy Cell Counter (Innovatis, Sittingbourne, UK). Each sample (cell suspension) was prepared three times in CAsyTon (Innovatis) buffer, followed by triplicate measurements of 200 μl sample volume. Viable cells were measured by Casy Cell Counter, by excluding all counts that were of a size smaller that 10 μm (dead cells and debris).

### Pharmacological treatments

CaCo2, HCT116, HT29 and SW480 cells were treated for 72 h with the indicated chemotherapeutic agents (10 uM 5FU and 10 uM OXA); alternatively, each cell line was treated 48 h after transfection with 1 μM, 5 μM, and 10 μM of 5FU (50 mg/ml concentrate, Sigma-Aldrich) or OXA (5 mg/ml concentrate, Sigma-Aldrich) and incubated for 48 h at 37 °C; three biological replicates were each assayed in triplicate and results were expressed as mean ± standard deviation (SD).

### Apoptosis assay by flow cytometry

Apoptosis was evaluated by AnnexinV-FITC and Propidium Iodide stained cells using Apoptosis Detection Kit (BD Biosciences), according to the manufacturer’s protocols. All flow cytometry results were analyzed with FACSuite Software v.1.0.5.3841 (BD Biosciences). Three biological replicates were prepared and each assayed in triplicate and the results expressed as mean ± standard deviation (SD).

### Cell-cycle analysis

Cell cycle analysis was performed three days after transfection on both attached and floating cells using the BD Cycletest Plus DNA reagent Kit (Cat. No. 340242; BD Biosciences). Propidium Iodide stained cells (>20.000 events) were analyzed by flow cytometry on FACSVerse (BD Biosciences). Debris and doublet cells were excluded and only single cells were considered for cell cycle analysis. Results were reported as percentage of cells in G1, S and G2/M phases. All flow cytometry results were analyzed with the FACSuite Software v.1.0.5.3841 (BD Biosciences). Four biological replicates were prepared and each assayed in triplicate; the results were reported as mean ± standard deviation (SD).

### Independent dataset analysis

The independent publicly available TCGA COAD series (https://tcga-data.nci.nih.gov/tcga/tcgaHome2.jsp) was analyzed to examine *CRY1* and *CRY2* expression in CRC tissues in comparison to normal mucosa. Normalized Level 3 data were used for our analysis. (Additional file 1: Figure S1)

### Statistical analysis

*CRY*1 and *CRY*2 expression levels in CRC tissue were compared with those of the adjacent non-tumorous mucosa, calculated using the formula 2-^ΔΔCt^ values and reported as median, 25^th^ percentile (or first quartile, Q1) and 75^th^ percentile (or third quartile, Q3). Where appropriate data of gene expression were log transformed to achieve normal distribution, verified by Normality test and Equal variance test. Hypotheses regarding differences among the values were compared by means of the paired *t*-test, Wilcoxon signed rank sum test, Student’s *t* test, Mann Whitney rank sum test. Pearson correlation coefficient *r* between gene expression levels was evaluated. Associations between gene expression level and phenotypic characteristics were evaluated by using the Pearson’s chi-squared test. Survival rates were calculated by the Kaplan–Meier method for analysis of censored data. Subgroup analyses were performed after splitting subjects at the median of gene expression levels or sorting them into quartile groups. For chronobiological analysis, after evaluation by Normality test and Equal variance test, time-effect across the time points and differences among the four cell lines at each time point were evaluated by ANOVA followed by all pairwise multiple comparison procedures (Student-Newman-Keuls Method). Each time-series of mRNA expression levels was analyzed for circadian rhythm characteristics by the single cosinor procedure involving the fit of a 24 h cosine curve to the data by least-squares linear regression, in order to accurately describe waveforms and rhythm characteristics. A *p* value for the rejection of the zero-amplitude assumption was determined for each component in the cosine model, with rhythm detection considered statistically significant if *p* < 0.05. Circadian characteristics were summarized from the 24 h cosine. Rhythm characteristics determined from the best-fitting cosine model included the MESOR (the middle of the cosine representing an adjusted average if unequal time interval), the amplitude, A (half the distance from the peak and trough of the best fitting curve), and the phase (Ø) of the cosine model (referenced to an external event, such as the serum shock), with the peak of a single component cosine called the acrophase, aØ (acro = peak). A p value <0.05 was considered statistically significant. All analyses were performed using the SPSS v17 Statistical Package (SPSS, Chicago, IL, USA) and the MATLAB statistical package (MathWorks, Natick, MA, USA).

## Results

### CRY1 and CRY2 mRNA levels are altered in colorectal cancer specimens

**Fig. 1:**
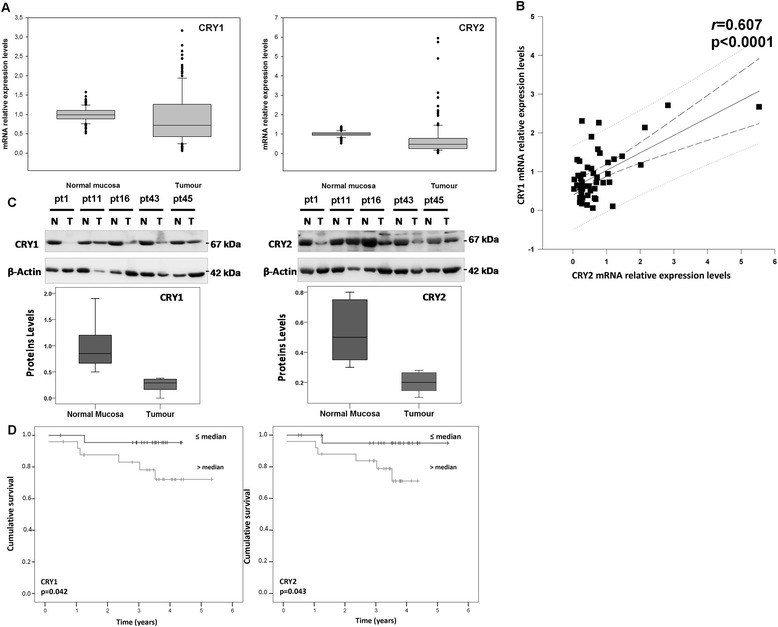
Expression of *CRY1* and *CRY2* mRNA and protein levels in colorectal cancer tissue and survival rates. **a** *CRY1* and *CRY2* mRNA levels analysed by qRT-PCR in colorectal cancer tissue and compared with matched normal tissue, with *GAPDH* expression used as the calibrator. A box and whisker plot is shown representing the interquartile range (IQR) with median, 25th and 75th percentile, minimum and maximum values, as well as outliers indicated by dots. **b** *x-y* plot showing regression lines with 95 % confidence limits between *CRY1* and *CRY2* mRNA expression levels in tumour tissues of CRC patients (n = 50, *r* = 0.607, *p* < 0.0001) **c**) CRY1 and CRY2 protein level evaluated by western blotting in a panel of matched specimens of tumour tissue and non-tumorous tissue. A box and whisker plot is shown representing the interquartile range (IQR) with median, 25th and 75th percentile, minimum and maximum values; **d**) Cumulative survival of CRC patients according to *CRY1* (left) and *CRY2* (right) expression levels. A significant difference was found in cumulative survival rates of CRC patients splitted at the median value according to *CRY1* expression (p = 0.042) and *CRY2* expression (p = 0.043). Patients with high expression levels showed significantly poorer survival rates

*CRY*1 and *CRY*2 mRNA levels were assessed in 50 pairs of tumour tissue/non-tumorous mucosa comprising 46 well-moderately differentiated (G1-G2) and 4 poorly differentiated-undifferentiated (G3-G4) CRCs. Median values, 25th and 75th percentile of *GAPDH*-Ct value/target gene-*C*_t_ value are shown in Fig. 1a. *CRY*1 (median = 0.73, Q1-Q3 = 0.43-1.24, *p* < 0.01) and *CRY*2 (median = 0.50, Q1-Q3 = 0.25-0.77, *p* < 0.001) mRNA levels showed a statistically significant decrease in tumour tissues in comparison to matched non-tumorous tissues (Fig. 1a). These results were validated by analyzing an independent publicly available cohort comprising 461 CRC patients (The Cancer Genome Atlas (TGCA) (https://tcga-data.nci.nih.gov/tcga/tcgaHome2.jsp) (Colon adenocarcinoma data, COAD) (Additional file 1: Figure S1). *CRY*1 and *CRY*2 mRNA expression correlated with each other in a Pearson’s correlation test (*r* =0.607, *p* < 0.0001) (Fig. 1b). Consistently, CRY1 and CRY2 protein levels decreased in tumour tissues compared to the non-tumorous counterpart in a panel of matched specimens (Fig. 1c).

### Association of CRY1 and CRY2 mRNA levels with patients and tumour characteristics

Genotype-phenotype associations were evaluated in our CRC patients cohort. After splitting subjects at the median value of gene expression levels, the decrease of *CRY1* mRNA levels observed in CRC tissue strongly associated with age, since the lowest expression levels were detected in patients with age range 62-74 years (*p* = 0.018), and with cancer location in the transverse colon (*p* = 0.008). Moreover, sorting subjects in quartile groups an association was found with gender, with the lowest levels detected in female patients (*p* = 0.003). No significant association was found between *CRY1* mRNA levels and grading (*p* = 0.778), modified Dukes stage (*p* = 0.929), histological type (*p* = 0.311) and MSI status (*p* = 0.659), (Table 2)

**Table 2:** Association of *Cry1* and *Cry2* mRNA levels with clinical and pathological features of colorectal cancer patients

			*Cry1*				*Cry2*			
		n = 50	Median	Q1	Q3	*P* value	Median	Q1	Q3	*P* value
Age	<62	16	1.067	0.727	1.315	0.018	0.661	0.318	0.920	0.408
	62-74	19	0.552	0.291	1.078		0.423	0.248	0.770	
	>74	15	0.715	0.329	0.943		0.434	0.211	0.742	
Gender	Male	34	0.879	0.427	1.398	0.003	0.432	0.236	0.801	0.677
	Female	16	0.686	0.442	0.819		0.551	0.297	0.673	
Tumor Location	Proximal colon^a^	17	0.722	0.323	1.078	0.008	0.434	0.254	0.742	0.007
	Transverse colon	6	0.546	0.389	0.649		0.232	0.127	0.309	
	Distal colon^b^	27	0.935	0.560	1.398		0.644	0.280	0.898	
Grading	G1/G2	45	0.726	0.427	1.174	0.778	0.477	0.248	0.801	0.925
	G3/G4	5	0.841	0.556	1.270		0.552	0.309	0.675	
Modified Dukes Staging System	A	4	0.612	0.419	0.912	0.929	0.326	0.203	0.831	0.397
	B	20	0.770	0.562	1.006		0.429	0.202	0.734	
	C	24	0.713	0.375	1.354		0.572	0.275	0.891	
	D	2	1.145	0.389	1.901		1.566	0.309	2.823	
Histologic Type	Non mucinous adenocarcinoma	45	0.318	0.291	0.552	0.311	0.592	0.270	0.607	0.083
	Mucinous adenocarcinoma	5	0.798	0.509	1.236		0.477	0.254	0.770	
MSI frequency	missing	12	-	-	-	0.659	-	-	-	0.555
	High	9	0,560	0,389	1,174		0,550	0,350	0,717	
	Low	10	0,770	0,583	1,110		0,403	0,248	0,675	
	Stable	19	0,628	0,318	1,078		0,592	0,270	1,043	
Tumor extent	2	4	0.612	0.419	0.912	0.582	0.326	0.203	0.831	0.625
	3	43	0.798	0.495	1.270		0.550	0.267	0.801	
	4	3	0.389	0.208	1.310		0.309	0.127	0.717	
Lymph node involvement	N0	24	0.727	0.502	1.006	0.205	0.438	0.230	0.785	0.482
	N1	14	0.569	0.318	1.270		0.419	0.269	0.715	
	N2	12	1.059	0.556	2.020		0.571	0.329	1.135	
TNM Stage	I-II	27	0.628	0.323	1.398	0.899	0.552	0.270	1.039	0.247
	III-IV	23	0.727	0.509	1.078		0.434	0.211	0.770	
Vascular Invasion	Not	27	0.727	0.509	1.110	0.620	0.552	0.267	0.801	0.621
	Yes	23	0.726	0.389	1.580		0.350	0.248	0.717	

^a^Caecum and ascending colon ^b^Descending colon, sigmoid colon and rectum

Likewise, lower *CRY*2 mRNA levels observed in CRC tissues strongly associated with cancer location, with the lowest levels detected in the transverse colon (*p* = 0.007). No significant association was found between *CRY*2 mRNA levels and age (*p* = 0.408), gender (*p* = 0.677), grading (*p* = 0.925), modified Dukes stage (*p* = 0.397), histological type (*p* = 0.083), and MSI status (*p* = 0.555) (Table 2).

After stratifying patients according to the median value of gene expression, subjects with *CRY*1 (*p* = 0.042) and *CRY*2 (*p* = 0.043) tumour mRNA levels above the median showed poorer survival rates in a Kaplan–Meier analysis of censored data (Fig. 1d). Sorting subjects in quartile groups the events were represented by one female subject out of 13 patients in the first quartile, one female subject out of 24 subjects in the second quartile and 5 male subjects out of 13 patients in the third quartile.

### Evaluation of CRY1 and CRY2 mRNA and protein levels in colon cancer cell lines

**Fig. 2:**
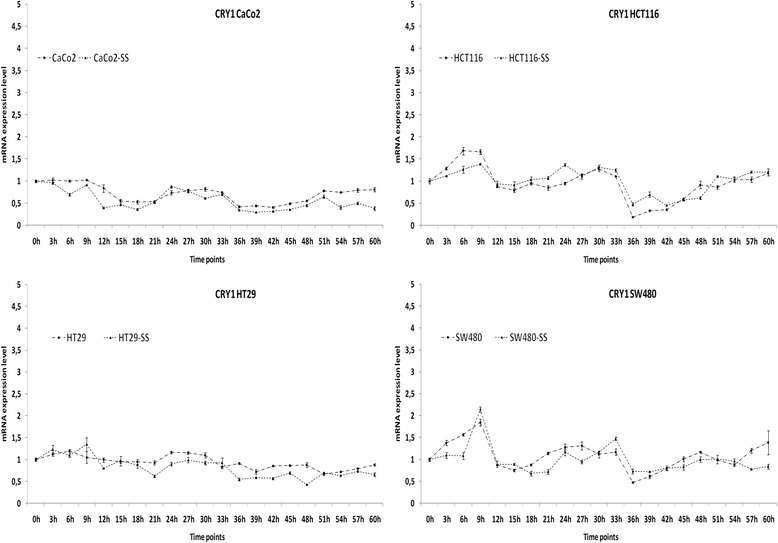
Time related patterns of cryptochrome gene expression in colon cancer cell lines. Relative expression of *CRY1* mRNA level in CaCo2, HCT116, HT29 and SW480 cells with and withour synchronization with serum shock (SS). Two biological replicates were each assayed in triplicate and results were expressed as mean ± standard deviation (SD)

**Fig. 3:**
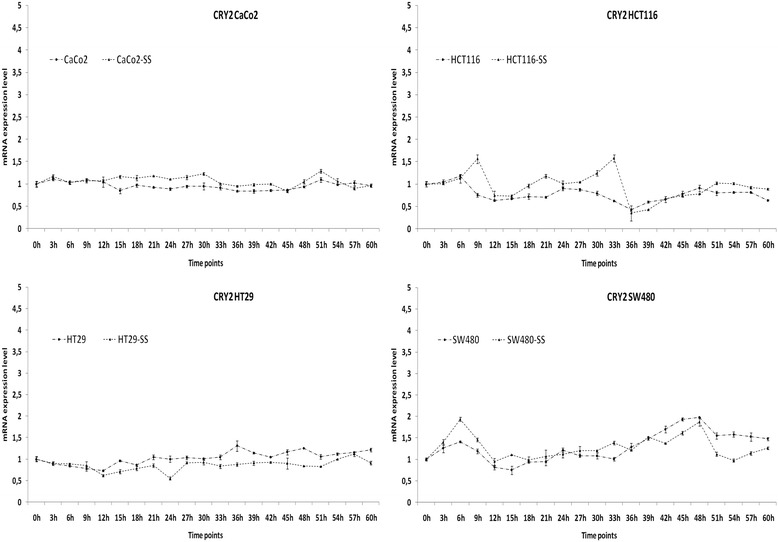
Time related patterns of cryptochrome gene expression in colon cancer cell lines. Relative expression of *CRY2* mRNA level in CaCo2, HCT116, HT29 and SW480 cells with and withour synchronization with serum shock (SS). Two biological replicates were each assayed in triplicate and results were expressed as mean ± standard deviation (SD)

The time related variation of cryptochrome gene expression was evaluated at 3-h intervals for sixty hours in four colon cancer cell lines with and without synchronization by serum shock. As shown in Figs. 2 and 3, we observed a time effect for the mRNA expression levels of *CRY1* and *CRY2* in CaCo2, HCT116, HT29 and SW480 cells, although circadian rhythmicity of transcription oscillation was evidenced only in HCT 116 cells. A significant difference was evidenced for *CRY1* and *CRY2* mRNA expression levels among the studied colon cancer cell lines at the examined time points when compared to non-tumorous mucosa; considering the harvesting time point at 21 h after synchronization, the highest *CRY1* expression levels were observed in CaCo2 and HCT116 cells respect to HT29 cells and especially to SW480 cells; the highest *CRY2* expression levels were observed in CaCo2 cells respect to HT29 and HCT116 cells and especially to SW480 cells (Additional file 2: Figure S2 and Additional file 3: Figure S3 and Table 3). Besides, the HCT116 cell line was hallmarked by higher *ARNTL* mRNA expression levels respect to CaCo2, HT29 and SW480 cells; HCT116 and HT29 cells showed the highest *c-MYC* mRNA expression levels and HT29 cells showed the highest *WEE* mRNA expression levels (Additional file 4: Figure S4 and Table 3).

**Table 3:** Summary of experimental data for the colon cancer cell lines examined

	CaCo2	HCT116	HT29	SW480
*CRY1* endogenous mRNA level^a^	↑↑↑	↑↑	↑↑	↑
*CRY2* endogenous mRNA level^a^	↑↑↑	↑↑	↑↑	↑
CRY1 endogenous protein level^b^	↑	↑↑↑	↑	↑↑
CRY2 endogenous protein level^b^	↑↑	↑↑↑	↑	↑
CRY1 ectopic protein level	↑	↑↑↑	↑	↑
CRY2 ectopic protein level	↑	↑↑↑	↑	↑
*TP53* status	mutated (Ø)	wild-type	mutated (GOF)	mutated (GOF)
p53 endogenous protein level	Ø	↑	↑↑↑	↑↑↑
p53 endogenous protein level upon *CRY1* ectopic expression	Ø	↑↑	=	=
p53 endogenous protein level upon *CRY2* ectopic expression	Ø	↑↑	↓	=
*ARNTL* endogenous mRNA level^a^	↑	↑↑↑	↑	↑
*c-MYC* endogenous mRNA level^a^	↑	↑↑↑	↑↑↑	↑
*WEE* endogenous mRNA level^a^	↑	↑↑	↑↑↑	↑↑
Cell pre-apoptosis upon *CRY1* ectopic expression	=	=	↓↓	↓↓
Cell pre-apoptosis upon *CRY2* ectopic expression	↓	=	↓	↓↓
Cell apoptosis upon *CRY1* ectopic expression	=	=	↓↓	↓↓
Cell apoptosis upon *CRY2* ectopic expression	↓	=	↓	↓↓
Cell viability upon *CRY1* ectopic expression	=	↑↑	↑↑	↑↑
Cell viability upon *CRY2* ectopic expression	↑	↑↑	↑↑	↑↑
Cell cycle changes upon *CRY1* ectopic expression	S↑ G2/M=	S↑ G2/M=	S↑ G2/M↓	S = G2/M↑
Cell cycle changes upon *CRY2* ectopic expression	S↑ G2/M=	S↑ G2/M=	S↑ G2/M↓	S↑ G2/M=
Cell pre-apoptosis upon treatment with OXA	↑↑↑	↑↑	↑↑	↑↑
Cell pre-apoptosis upon treatment with 5FU	↑↑↑	=	↑↑	↑↑
Cell apoptosis upon treatment with OXA	↑↑↑	↑↑	↑↑	↑↑
Cell apoptosis upon treatment with 5FU	↑↑↑	=	↑↑	↑↑
Cell viability upon *CRY1* ectopic expression + OXA^c^	↓	↓	=	↓
Cell viability upon *CRY2* ectopic expression + OXA^c^	↓	=	=	↓
Cell viability upon *CRY1* ectopic expression + 5FU^c^	=	↑	↓	↑
Cell viability upon *CRY2* ectopic expression + 5FU^c^	=	↑	=	↑

^a^cells harvested 21 h after synchronization with serum shock; ^b^cells harvested 22 h after synchronization with serum shock; *Ø* protein not detected, *GOF* gain of function; ^c^compared to mock transfected cells

**Fig. 4:**
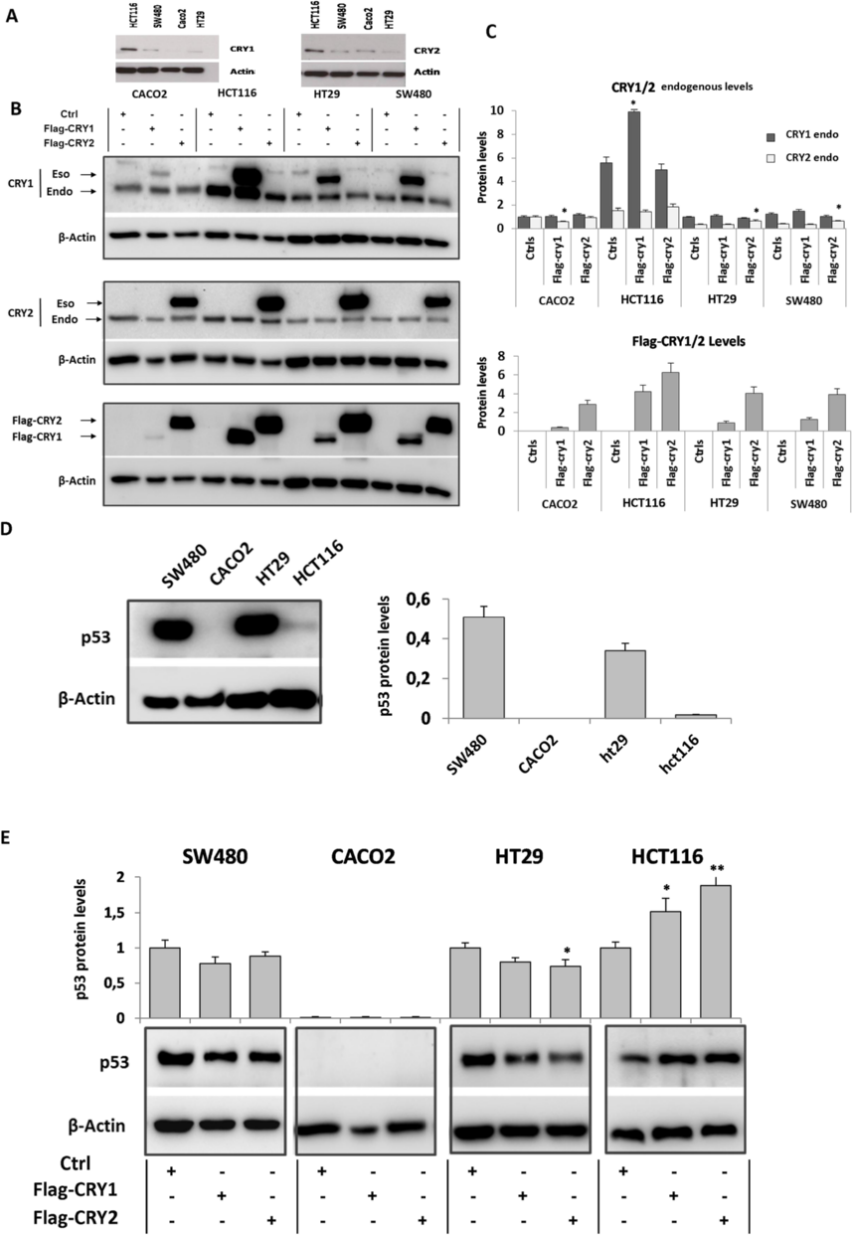
Evaluation of CRY1 and CRY2 protein levels in colon cancer cell lines. **a** Immunoblot detection of CRY1 and CRY2 protein in CaCo2, HCT116, HT29 and SW480 cells harvested 22 h after synchronization with serum shock; **b**-**c**) Immunoblot detection of CRY1 and CRY2 protein in CaCo2, HCT116, HT29 and SW480 cells with and without cryptochrome gene ectopic expression. Western blot analysis was performed to detect the protein expression levels of CRY1 and CRY2 upon transfection using anti-CRY1 and anti-CRY2 antibody as well as anti-FLAG antibody. β-Actin antibody was used as control. **d** basal p53 levels in CaCo2, HCT116, HT29 and SW480 cells; **e**) p53 levels upon *CRY1* and *CRY2* ectopic expression in the four colon cancer cell lines; *bars*, standard deviation (SD); *P < 0.05; **P < 0.01. Each western blot analysis was performed five times

Western blot analysis performed on extracts of cells harvested 22 h after synchronization showed higher levels of CRY1 and CRY2 proteins in HCT116 cells compared to the other cell lines examined (Fig. 4a). All four cell lines were then transiently transfected with expression vectors carrying *CRY1* and *CRY2* cDNAs fused to the FLAG epitope at their 5’ end and the resulting proteins assessed by western blot. The exogenous proteins, detected by a FLAG specific antibody, exhibited variable levels likely due to a different transfection efficiency; they were higher in HCT116 than SW480 and HT29 than Caco2 cells as shown in the relative histogram (*p* < 0.05) (Fig. 4b and c). Higher p53 protein levels were found in HT29 and SW480 cells respect to HCT116, whereas it was not detectable in CaCo2 cells (Fig. 4d). Protein levels of p53 increased in HCT116 cells upon *CRY1* and *CRY2* ectopic expression (*p* < 0.05 and *p* < 0.01), while decreased in HT29 cells upon *CRY2* ectopic expression (*p* < 0.05) (Fig. 4e).

### FISH comparative analysis of CRY1 and CRY2 copy numbers in colon cancer cell lines

**Fig. 5:**
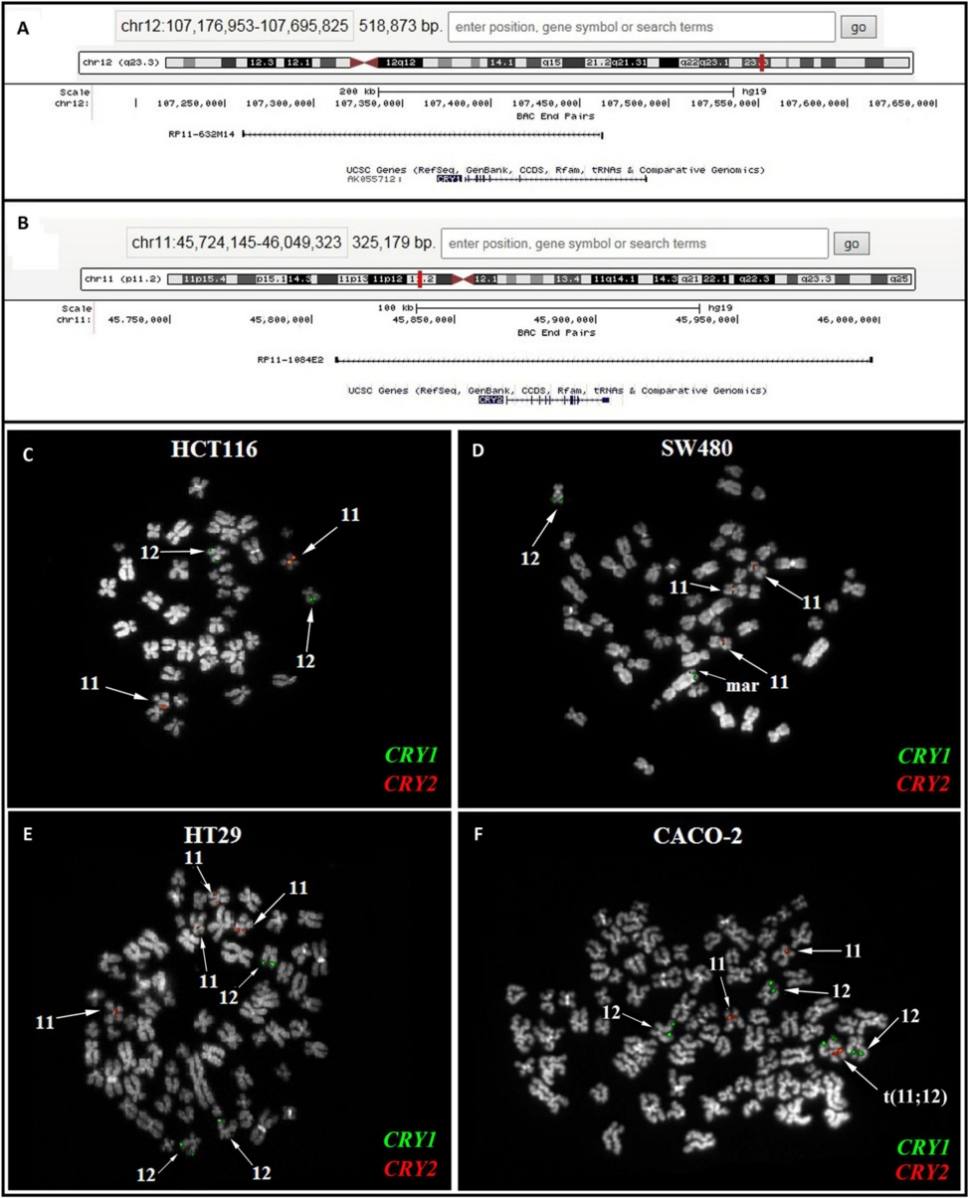
FISH experiments performed on colon cancer cell lines. **a**-**b** UCSC maps of the BAC clones used for *CRY1* (**a**) and *CRY2* (**b**) copy number alterations. Genes are reported in blue at the bottom of the figures. **c**-**f** FISH results for *CRY1* and *CRY2* probes (in green and red, respectively) obtained in HCT116 (**c**), SW480 (**d**); HT29 (**e**), and CACO-2 (**f**) cells. See text for details

The occurrence of copy number variations involving *CRY1* and *CRY2* genes was assessed by Fluorescence In Situ Hybridization (FISH) on the studied CRC cell lines employing specific probes of chromosome 12 and 11 where *CRY1* and *CRY2* genes are respectively located. The *CRY1* gene displayed a low copy number gain (>2 spots/probe) in HT29 and CaCo2 cells, both having a whole-chromosome 12 trisomy (Fig. 5e); a marker chromosome carrying the *CRY1* signal was also detected in CaCo2 cells (Fig. 5 f). Conversely, *CRY1* displayed two spots in HCT116 and SW480 cells, one of which was on a normal chromosome 12, while the second on a translocated one (Fig. 5c and d). Similarly, *CRY2* disclosed a low copy number gain in SW480 and HT29 cells, represented by a chromosome 11 trisomy and tetrasomy, respectively (Fig. 5d and e); in CaCo2 cells an additional signal on a translocated chromosome 11 was detected (Fig. 5 f and e). Conversely, in HCT116 cells, *CRY2* displayed only the two normal chromosomes 11 signals (Fig. 5c).

### Comparative analysis of apoptosis, proliferation, cell cycle upon cryptochrome genes ectopic expression in colon cancer cell lines

**Fig. 6:**
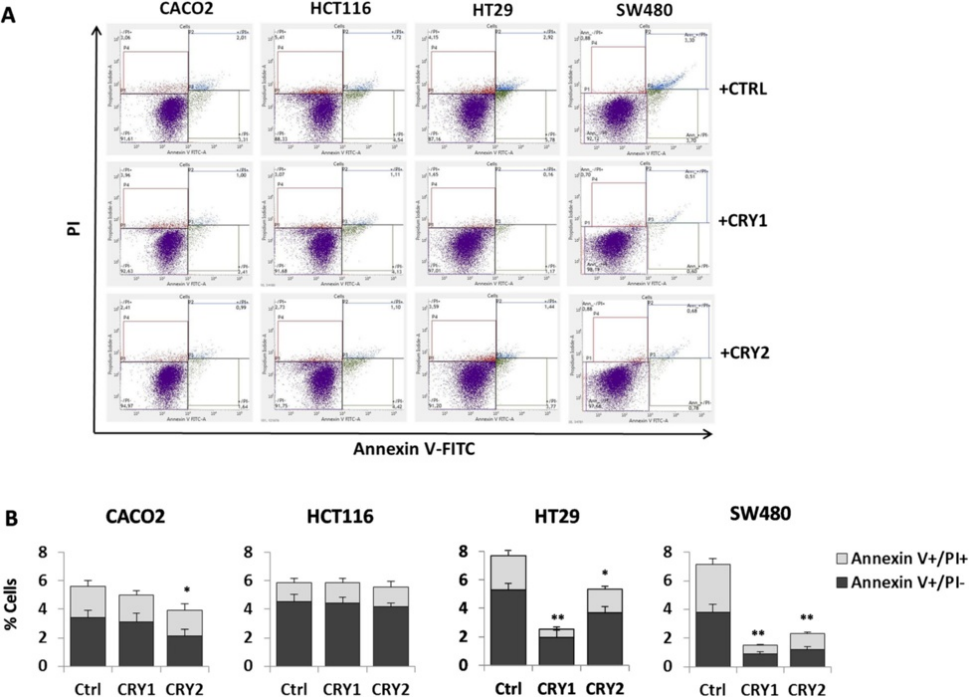
Evaluation of apoptosis changes upon *CRY1* and *CRY2* ectopic transfection in colon cancer cell lines. **a-b** Apoptosis was evaluated by AnnexinV-FITC and Propidium Iodide stained cells using Apoptosis Detection Kit (BD Biosciences), according to the manufacturer’s protocols. All flow cytometry results were analyzed with FACSuite Software v.1.0.5.3841 (BD Biosciences); *bars*, standard deviation (SD); *P < 0.05; **P < 0.01; three biological replicates were prepared and each assayed in triplicate and the results expressed as mean ± standard deviation (SD)

**Fig. 7:**
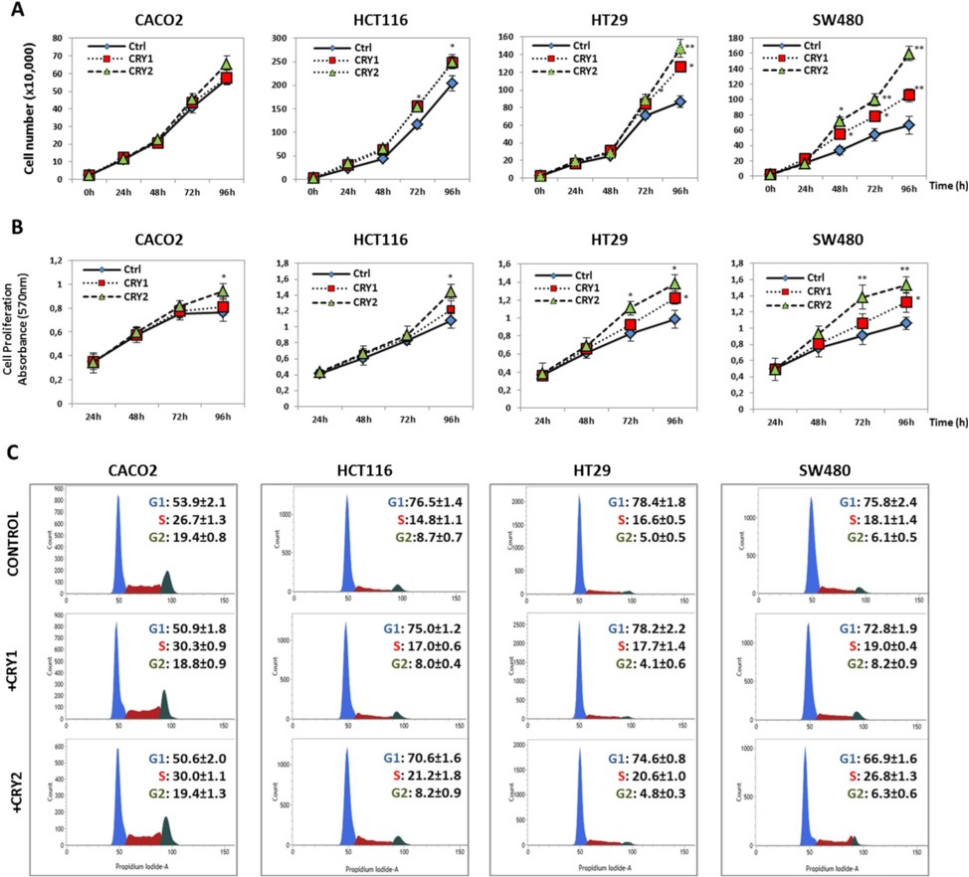
Evaluation of proliferation and cell cycle changes upon *CRY1* and *CRY2* ectopic expression in colon cancer cell lines. **a** MTT assays performed on CaCo2, HCT116, HT29 and SW480 cells transfected with *CRY1* or *CRY2* constructs. Wells containing transfected cells with reagents alone (Mock) were used as negative control; **b**) cell counts performed on CaCo2, HCT116, HT29 and SW480 cells transfected with *CRY1* or *CRY2* constructs. Wells containing transfected cells with reagents alone (Mock) were used as negative control; three biological replicates were prepared and each assayed in triplicate; the results were reported as mean ± standard deviation (SD); **c**) cell cycle analysis was performed three days after transfection on both attached and floating cells using the Cell-Cycle Test (BD Biosciences). Propidium Iodide stained cells (>20.000 events) were analyzed on FACSVerse flow cytometer (BD Biosciences); *bars*, standard deviation (SD); *P < 0.05; **P < 0.01; four biological replicates were each assayed in triplicate and results were expressed as mean ± SD

Data from our tumour specimens led us to hypothesize that variations in cryptochrome genes expression might control the transformed cell phenotype. To verify this, we sought to test the effects on apoptosis and cell proliferation of ectopic CRY1 and CRY2 proteins in CRC cells (see Figs. 6a and b and 7a and b). Exogenous expression of both proteins in the HT29 and SW480 cell lines induced a statistically significant reduction of apoptotic and pre-apoptotic cells by flow cytometry. Specifically, the induction was stronger with CRY1 than CRY2 although the transfection efficiency was lower for the former protein, suggesting a robust effect on apoptosis. Milder effects were detected in CaCo2 cells for both proteins, while no variations were detected in HCT116 cells, albeit the large amount of the transfected FLAG-tagged proteins (Fig. 4a and b). In addition, we evaluated cell proliferation at different time points from transfection and found that HT29 and SW480 cells in particular, exhibited a higher proliferation rate than CaCo2 and HCT116 cells (Fig. 7a and b). Concordant results were obtained with two independent methods such as automatic cell counting and MTT assay. When the cell cycle effects were monitored by flow cytometry, exogenous CRY2 induced an increase of the S phase in all cell lines with a concomitant reduction of the G1 phase cell population; in contrast, CRY1 produced a less evident effect (Fig. 7c).

Data from the literature have reported that the *TP53* status influences *CRY1* and *CRY2* response in vivo. We assessed basal levels of p53 in the four cell lines investigated and the effects of exogenous *CRY1* and *CRY2* on p53 protein levels. Upon *CRY* expression constructs transfection, SW480 and HT29 cells, which exhibit high levels of a mutated p53 protein (R273H), displayed only a slight reduction. CACO2 cells showed no p53 protein due to a truncating mutation (G204X), while HCT116 cells exhibited an increase of the wild type p53 protein (Fig. 4e).

### Evaluation of response to chemotherapeutic agents upon cryptochrome genes ectopic expression in colon cancer cell lines

Having shown that cryptochrome genes expression affects cell death and proliferation, we sought to verify whether they might influence the response to chemotherapeutic agents in colon cancer cells. To this aim, we evaluated changes in apoptosis/cell viability upon exposure to selected drugs in basal conditions and after ectopic cryptochrome gene expression.

**Fig. 8:**
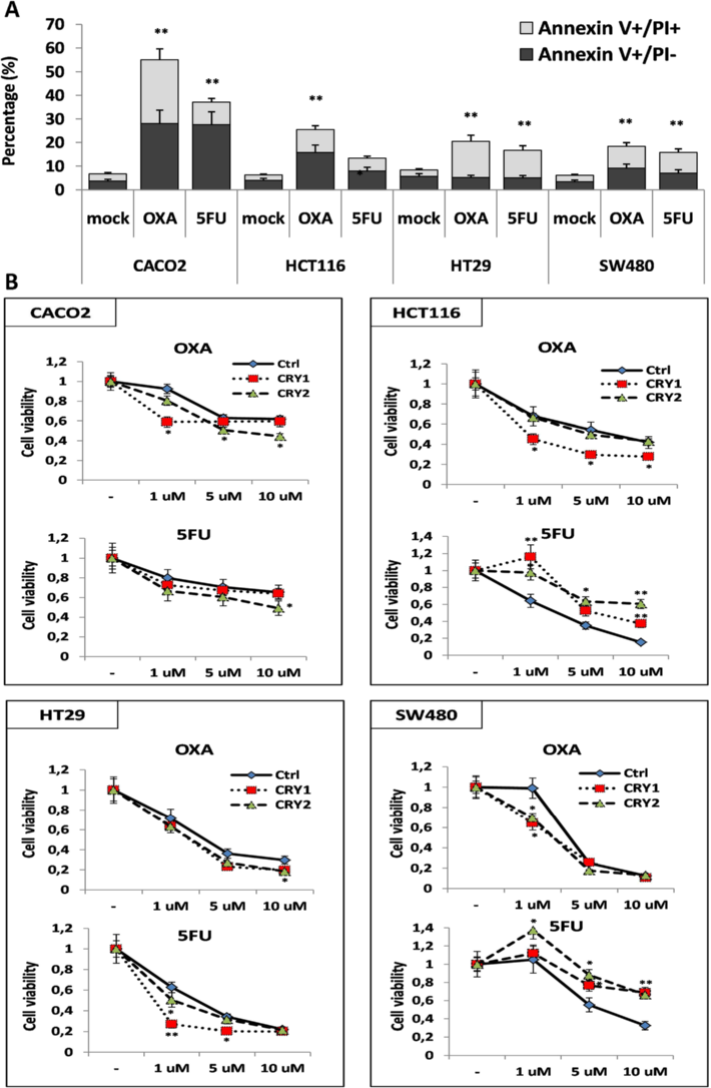
Patterns of response to chemotherapeutic agents in colon cancer cell lines. **a** apoptotic and pre-apoptotic response to treatment with 5-fluorouracil (5-FU) or oxaliplatin (OXA) in CaCo2, HCT116, HT29 and SW480 cells; **b**) Effect of *CRY1* or *CRY2* ectopic expression on response to treatment with 5FU or OXA in colon cancer cell lines. The cell lines were treated with 1 μM, 5 μM and 10 μM of 5FU or OXA for 24 h at 37 °C in 5 % CO_2_ atmosphere 48 h after transfection with *CRY1* or *CRY2* constructs. Wells containing transfected cells with *CRY1* or *CRY2* constructs were used as negative control (Mock). Cell viability was determined by MTT assay. *bars*, standard deviation (SD); *P < 0.05; **P < 0.01; three biological replicates were each assayed in triplicate and results were expressed as mean ± SD

A variable response to 10 uM 5FU and 10 uM OXA was detected in all four cell lines, with Caco2 cells being the most responsive ones (Fig. 8a).

CRY1 ectopic expression in CaCo2 cells did not influence the cytopathic effect upon treatment with 5FU while increased that of 1 μM OXA (*p* < 0.05); CRY2 ectopic expression increased the cytopathic effect upon treatment with 10 μM 5FU (*p* < 0.05) and 5 μM and 10 μM OXA (*p* < 0.05). In HCT116 *CRY1* ectopic expression decreased the cytopathic effect upon treatment with 1, 5 and 10 μM 5FU (*p* < 0.05) and increased that of 1, 5 and 10 μM OXA (*p* < 0.05 and *p* < 0.01 respectively). On the other hand, *CRY2* ectopic expression decreased the cytopathic effect upon treatment with 1, 5 and 10 μM 5FU (*p* < 0.05) and did not influence the effect of OXA. In HT29 cells *CRY1* ectopic expression increased the cytopathic effect upon treatment with 1 μM and 5 μM 5FU (*p* < 0.05) and 10 μM OXA (*p* < 0.05), whereas CRY2 ectopic expression increased the cytopathic effect upon treatment with 1 μM 5FU (*p* < 0.05) and 10 μM OXA (*p* < 0.05). In SW480 cells *CRY1* and *CRY2* ectopic expression decreased the cytopathic effect upon treatment with 5FU (*p* < 0.05) and increased the cytopathic effect upon treatment with 1 μM OXA (*p* < 0.05) (Fig. 8b).

## Discussion

In the present study, we evaluated the expression of cryptochrome genes in tumour tissues of a large cohort of CRC patients and explored their expression patterns in synchronized colon cancer cell lines. *CRY1* and *CRY2* mRNA levels were significantly changed in CRC tissue when compared to matched non-tumorous mucosa, and there was a positive linear correlation between mRNA expression levels of both cryptochrome genes, probably indicating a corresponding and parallel alteration in tumour tissues. These data broaden the results obtained in a previous study [25] and were validated by an independent publicly available dataset (461 CRC patients of The Cancer Genome Atlas (TGCA) cohort). Moreover, the observed *CRY1* mRNA levels strongly correlated with age, being the lowest levels detected in the group of patients 62–74 years of age. Epidemiological data report higher incidence of CRC in the elderly, with 80-90 % of cases arising in people who are in the sixties or older and the median age at presentation is 72 years. Up to 50 years of age, men and women exibit similar rates for bowel cancer, but at older ages male rate predominates, and the lifetime risk of being diagnosed with CRC is reported to be 5.9 % for men and 5.5 % for women [16, 17]. Additionally, lower *CRY1* mRNA levels were found in female CRC patients, while higher *CRY1* and *CRY2* levels were observed in tumours located at the distal colonic segments (descending and sigmoid colon, rectum). In contrast, lower *CRY1* and *CRY2* expression was found in tumour tissues located at the transverse colon. Epidemiological data report that 70 % of new CRCs occurs in the colon (25 % in the sigmoid colon), while the remaining 30 % arises in the rectum; the transverse colon is a relatively less frequent tumour location, although more frequent in female patients [16, 17]. These data support our present evidence of an association between female gender, tumour location at the transverse colon and lower expression of cryptochrome genes. In mammals, xenobiotic detoxification is a clock controlled function, with hepatic, intestinal and renal detoxification systems showing circadian variations of their ability to inactivate noxious agents, determining time-dependent activity and toxicity of drug administration [29]. Chronomodulated chemotherapy has shown better tolerability and antitumour activity with respect to conventional chemotherapy, with less mielosuppression in spite of more gastrointestinal toxicity, but no difference in median survival time. A gender related difference was observed, with a median survival with chronotherapy approximately 5 months longer in male CRC patients and greater incidence of severe toxicities in female CRC patients [30].

From these studies gender comes out as the single predictor of survival, conditioning the outcome of chronoFLO4 and determining genetic variation of metabolic responses that influence time-related variables and hinder administration of maximal effective dosing. A different genotypic profile between males and females could characterize CRC patients and the higher burden of toxicity reported in women treated with fluorouracil-based chemotherapy may be also due to the lower expression of dihydropyrimidine dehydrogenase (*DPD*) found in the tumours of female patients and/or to the gender dependency of circadian pharmacology [31]. Translational studies of circadian genes that influence pharmacokinetics, pharmacodynamics, and drug metabolism may highlight these critical issues and may point out the molecular biomarkers that could drive optimal timing of chronochemotherapy delivery schedules.

Patients bearing higher *CRY*1 and *CRY2* expression exhibited a poorer survival in Kaplan-Meier survival curves. These data corroborate the reported lower overall and disease free survival rate related to higher *CRY1* expression [32], and are supported by epidemiological data evidencing that CRC related mortality rates are higher in men than in women [16, 17].

Evaluation of time related pattern of cryptochrome genes expression in the CaCo2, HCT116, HT29 and SW480 cell lines after synchronization with serum shock produced variable results: *CRY1* and *CRY2* mRNAs changed in a time related manner in all the cell lines examined and showed 24-h periodicity of variation only in HCT116 cells. These results might be related to the neoplastic nature of the examined cell lines and might be interpreted taking into account previous studies that assessed clock gene expression in the mouse gastrointestinal mucosa showing circadian rhythmicity only in some studies for *CRY1* and no oscillation for *CRY2* [3, 33–35].

Pinpointing cryptochrome gene expression in cells harvested 21 h after serum shock induced synchronization, CaCo2, HCT116 and HT29cells showed higher *CRY1* and *CRY2* mRNA levels compared to SW480 cells, whereas the SW480 cell line showed higher *CRY1* levels at several time points considering not synchronized cells. Our results are in agreement with a previous report showing higher *CRY1* expression in SW480 cell, disclosing high proliferation rate and invasiveness [32]. Of note, our parameters were appraised after cell synchronization to obtain more valuable and reliable results. Furthermore, we did not find a direct correlation between *CRY1* and *CRY2* mRNA and the corresponding protein levels, evaluated in cells harvested 22 h after synchronization, likely due to the gap between gene transcription and protein synthesis or tagging and degradation processes. We also carried out FISH experiments to verify whether the different cryptochrome genes expression in the studied cell lines could be related to gene copy number variations. A low copy number gain was detected for *CRY1* in HT29 and CaCo2 cell lines and for *CRY2* in SW480 and HT29 cells, suggesting that copy number gain does not fully explain the differences in gene expression observed and that epigenetic mechanisms might likely be involved.

FISH and immunoblotting experiments thus suggest that cryptochrome gene expression could be post-transcriptionally and post-translationally regulated in the colon cancer cell lines examined.

Ectopic expression of *CRY1* and *CRY2* decreased apoptosis/preapoptosis in CaCo2, HT29 and SW480 cells, increased proliferation rate and percentage of cells in S phase in the four colon cancer cell lines examined, corroborating the evidence of poorer prognosis observed in CRC patients showing higher expression of cryptochrome genes in their tumour tissues.

A number of drugs can be employed for CRC chemotherapy and frequently, a combination of two or more of these drugs is more effective. A common drug combination used for adjuvant treatment include OXA and 5FU, which is often given with folinic acid, which enhances its action. The Challenging with OXA and 5FU the examined colon cancer cell lines evidenced different changes of cytopathic effects in the colon cancer cell lines studied after each cryptochrome gene transfection. Interestingly, recent studies evidenced that CRY1 modulates the ATR-mediated DNA damage checkpoint response by interacting in a time-of-day-dependent manner with TIMELESS, another protein involved in the molecular clockwork in addition to the DNA damage response, and CRY2 takes part in the regulation of DNA damage repair as well as the maintenance of genomic stability [36, 37].

The genotypic-phenotypic associations and effects on overall survival of cryptochrome genes reported in our CRC patients and the different *CRY1* and *CRY2* expression levels and time related profiles plus the different apoptotic, proliferative and cytotoxic responses observed in vitro in the studied colon cancer cell lines could be related to the dissimilarity of their chromosomal abnormalities and genetic background. In some way, these data may suggest a molecular pathophysiological mechanism causing the differences in disease behavior and response to therapy observed in vivo in CRC patients. In this respect, a crucial factor could be represented by p53 status. The relationship with p53 status has been proposed as an additional layer of regulation in vivo. Indeed, analysis of the chosen colon cancer cell lines showed that higher CRY1 and CRY2 protein levels coincided with a wild type p53 as in HCT116 cells and that this condition only marginally affected the apoptotic and cell proliferation characteristics of the cells upon ectopic expression. In contrast, lower CRY and CRY2 levels as in HT29 and SW480 cells were associated with a mutated p53 and a more robust apoptosis and proliferation upon transfection. Overall, the phenotypic differences among the colon cancer cell lines investigated may underlie diverse *CRY*1 and *CRY*2 expression patterns and a related genetic landscape that could influence cell survival and response to chemotherapy. The gene coding for p53 (*TP53*) is expected to be mutated in 40–50 % of CRCs, and our colon cancer cell lines carried different *TP53* mutations and showed variable expression of p53 protein [38]. Particularly, CaCo2 cells show a *TP53* point mutation determining undetectable p53 protein by immunohistochemistry, HCT116 cells do not show *TP53* mutation, HT29 and SW480 cells show *TP53* point mutation, p53R273H and p53R273H/P309S respectively, which induces the mutated proteins to attain ‘gain of function’ (GOF), dynamically involved in cancer development and progression [39]. The interplay between cryptochrome genes and p53 has been extensively studied in mouse models. In *Cry1-/-Cry2-/-* mutant mice radiation-induced morbidity and mortality are similar to that observed in the wild-type controls. In the same way, the DNA damage checkpoint response to ionizing radiation of *Cry1-/-Cry2-/-* mutant fibroblasts is comparable to that of the wild-type controls, suggesting that cryptochrome genes and biological clock disruption for itself is not capable to prejudice mammalian DNA damage checkpoints and repair [40]. On the contrary, the *p53-/-Cry1-/-Cry2-/-* mice show delayed spontaneous carcinogenesis when compared to *p53-/-* mice, suggesting that *Cry* mutation may activate p53-independent apoptosis pathways. Indeed, cell lines generated from the *p53-/-Cry1-/-Cry2-/-* mice are more susceptible to UV-induced apoptosis when challenged with genotoxic stress respect to *p53-/-* cells in the presence of preserved DNA damage checkpoint functions and repair [41]. When tumours arising from oncogenic Ras-transformed *p53-/-* and *p53-/-Cry1-/-Cry2-/-* cells are treated with OXA, *p53−/−* tumours continue to grow whereas *p53-/-Cry1-/-Cry2-/-* tumours exhibit extensive apoptosis and stop growing, corroborating the evidence that cryptochrome disruption in *p53-/-* cells makes them more sensitive to chemotherapy by OXA [42]. Essentially, cryptochrome gene mutation hampers tumorigenesis in *p53-/-* mice by means of: *(i)* stimulation of p53-independent apoptosis pathways, *(ii)* reduction of antiapoptotic action of NF-κB signaling upon inflammatory cytokine stimulation, *(iii)* purging of premalignant and malignant cells with hindrance of overt tumour formation [43]. In the presence of DNA damage, and with the aim to repair DNA alterations and prevent accumulation of mutations in the genome of daughter cells, normal human cells arrest either in G1 or S phase of the cell cycle. The G1 phase cell cycle arrest relies on p53 and is mainly accomplished by the cyclin-dependent kinase (Cdk) inhibitor p21^(Cip1/Waf1)^. The signaling pathway dependent on p53 is modulated by the circadian protein ARNTL and in particular ARNTL-silenced cells are not capable to arrest upon p53 activation due to failure to activate the p53 target gene p21^(Cip1/Waf1)^. ARNTL is required for the p53-dependent induction of p21^(Cip1/Waf1)^ and ARNTL suppression affects the ability of p53 to induce cell cycle arrest upon cellular stress signals such as DNA damage [44]. ARNTL restrains the G2/M transition by inducing the expression of WEE kinase that inhibits Cdk1, and inhibits p21^(Cip1/Waf1)^ expression thus triggering Cdk2 and Cdk1, which prop up the S and M phases; ARNTL also inhibits c-MYC and thus decreases the expression of cyclin E and the activity of cyclin E/Cdk2, hindering the G1/S transition [45]. Interestingly, WEE kinase is elevated in *Cry* double-mutant mouse fibroblasts [46], but their growth rate does not differ from the wild-type controls, likely through compensation by faster progression through other cell cycle phases [47]. Based on these premises, the different phenotypic hallmarks and patterns of response to chemotherapeutic agents observed in the colon cancer cell lines examined upon *CRY1* and *CRY2* ectopic expression must be interpreted taking into account the interplay between the cryptochrome genes and the genetic landscape of the neoplastic cells. Accordingly, pinpointing two opposite paradigmatic cell models, we observed increased sensitivity to OXA in CaCo2 cells, which do not express p53 protein and have low *CRY1*, *CRY2*, *ARNTL*, *c-MYC* and *WEE* expression levels, whereas no change in sensitivity was shown in HT29 cells, which are characterized by GOF of mutated *TP53*, by low *CRY1, CRY2* and *ARNTL* expression levels, but high *c-MYC* and *WEE* expression levels. Interestingly, severely decreased effect of 5FU upon ectopic cryptochrome gene expression was evidenced in the HCT116 cell line, which is characterized by high endogenous cryptochrome gene expression at mRNA and protein level and shows increase of p53 levels upon *CRY1* and *CRY2* ectopic expression. In view of the translational and clinical implications, we must consider that cryptochrome and *ARNTL* gene expression levels in the tumour tissue of CRC patients influence chemotherapy response and survival. CRY2 over-expression in human CRC samples may be caused by down-regulation of *FBXW7*, encoding F-box and WD repeat domain containing 7, E3 ubiquitin protein ligase, which binds directly to phosphorylated Thr300 of CRY2 and tags this circadian protein for proteasomal degradation. High CRY2 and low FBXW7 expression in colorectal tumour tissue was correlated with chemoresistance as well as poorer survival of CRC patients [48]. Besides, in vitro and in vivo experiments showed that *ARNTL* over-expression inhibited colon cancer cell proliferation and increased CRC sensitivity to OXA. Besides, high *ARNTL* expression level in primary tumour specimens of CRC patients treated with OXA based regimens (FOLFOX or XELOX) was associated with significantly longer overall and progression-free survival respect to patients with low *ARNTL* levels. The molecular mechanism is related to *ARNTL* control of G2/M arrest through ATM pathway activation [49].

## Conclusions

In conclusion, in malignant colorectal neoplastic disease the expression of cryptochrome genes is severely altered, particularly in elderly subjects, female patients and cancers located in the transverse colon. On the other side, lower expression levels in tumour tissue seem to predict better survival in CRC patients. The different *CRY1* and *CRY2* expression levels and time related profiles plus the different apoptotic, proliferative and cytotoxic responses observed in vitro in the studied colon cancer cell lines could be related to the dissimilarity in their chromosomal abnormalities and genetic background. The interplay between cryptochrome genes and different genomic landscapes may influence cancer cell phenotype, impinging on neoplastic disease behavior and response to therapy, possibly modifying sex dimorphism-related differences in drug toxicity and outcome measures.

## Additional files

Additional file 1: Figure S1. Expression of *CRY1* and *CRY2* mRNA levels in colorectal cancer tissue of 461 patients from the TGCA cohort (https://tcga-data.nci.nih.gov/tcga/tcgaHome2.jsp). A box plot is shown representing the interquartile range (IQR) with median, 25th and 75th percentile, minimum and maximum values, as well as each outlier indicated by dots and stars. (JPG 247 kb) Additional file 2: Figure S2.
*x-y* plots representing the time related mRNA expression profiles of *CRY1* in CaCo2, HCT116, HT29 and SW480 cells with and without synchronization with serum shock and normalized to non-tumorous colorectal mucosa. Two biological replicates were each assayed in triplicate and results were expressed as mean ± standard deviation (SD). (JPG 652 kb) Additional file 3: Figure S3.
*x-y* plots representing the time related mRNA expression profiles of *CRY1* in CaCo2, HCT116, HT29 and SW480 cells with and without synchronization with serum shock and normalized to non-tumorous colorectal mucosa. Two biological replicates were each assayed in triplicate and results were expressed as mean ± standard deviation (SD). (JPG 568 kb) Additional file 4: Figure S4. A) *x-y* plots representing the time related mRNA expression profiles of *ARNTL*, *WEE* and *c-MYC* in CaCo2, HCT116, HT29 and SW480 cells synchronized after serum shock and harvested at the indicate time points. Two biological replicates were each assayed in triplicate and results were expressed as mean ± standard deviation (SD). B) ARNTL, WEE and c-MYC protein level evaluated by western blotting in a panel of matched specimens of tumour tissue and non-tumorous tissue. (JPG 473 kb)
