# Supplementary figures and images for: Deregulated expression of cryptochrome genes in human colorectal cancer
Source: Mol Cancer. 2016 Jan 15;15:6. doi: 10.1186/s12943-016-0492-8 (PMC4714521; doi:10.1186/s12943-016-0492-8)

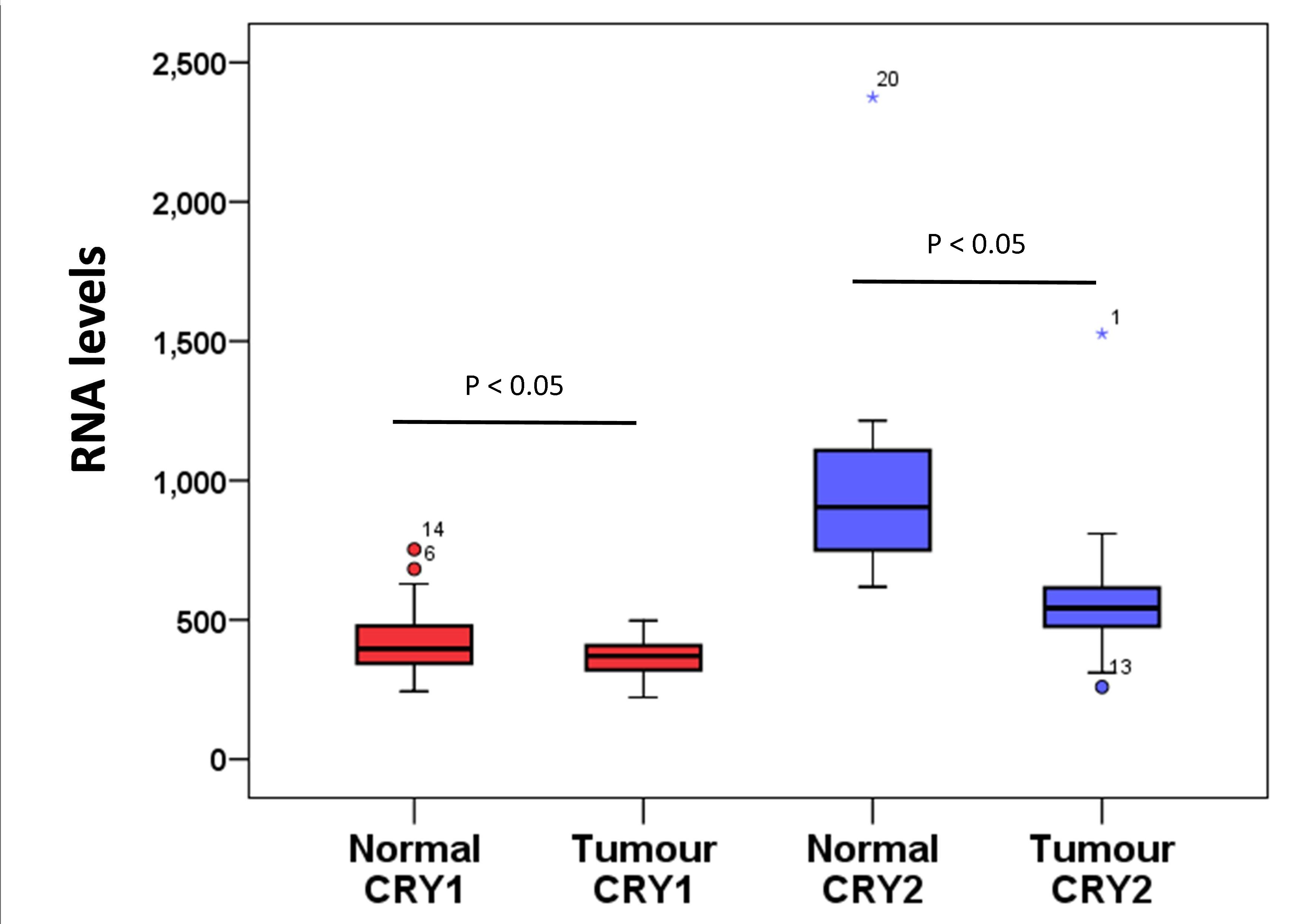

Supplement: Additional file 1: Figure S1. — Expression of CRY1 and CRY2 mRNA levels in colorectal cancer tissue of 461 patients from the TGCA cohort (https://tcga-data.nci.nih.gov/tcga/tcgaHome2.jsp). A box plot is shown representing the interquartile range (IQR) with median, 25th and 75th percentile, minimum and maximum values, as well as each outlier indicated by dots and stars. (JPG 247 kb) [file 12943_2016_492_MOESM1_ESM.jpg]

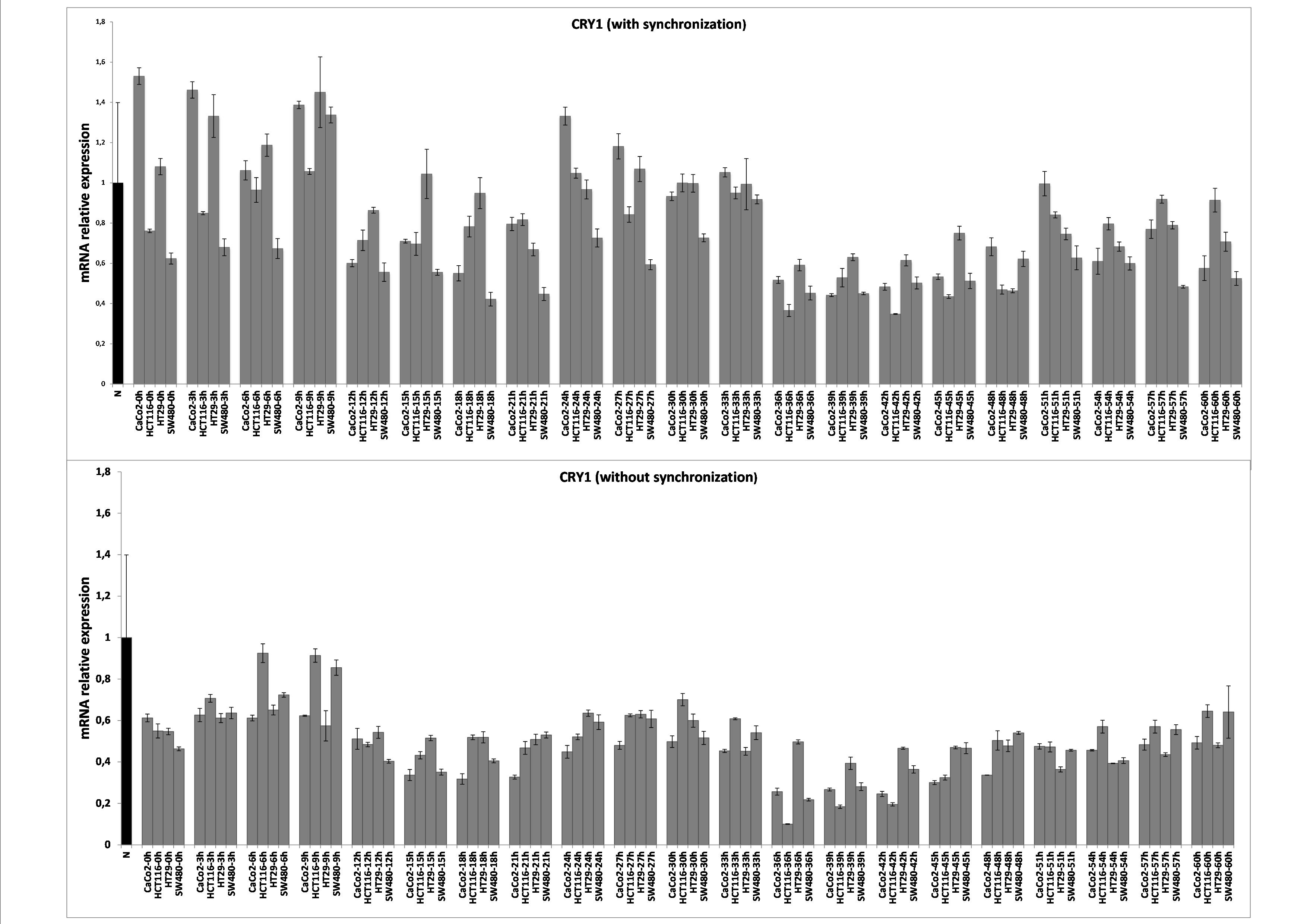

Supplement: Additional file 2: Figure S2. — x-y plots representing the time related mRNA expression profiles of CRY1 in CaCo2, HCT116, HT29 and SW480 cells with and without synchronization with serum shock and normalized to non-tumorous colorectal mucosa. Two biological replicates were each assayed in triplicate and results were expressed as mean ± standard deviation (SD). (JPG 652 kb) [file 12943_2016_492_MOESM2_ESM.jpg]

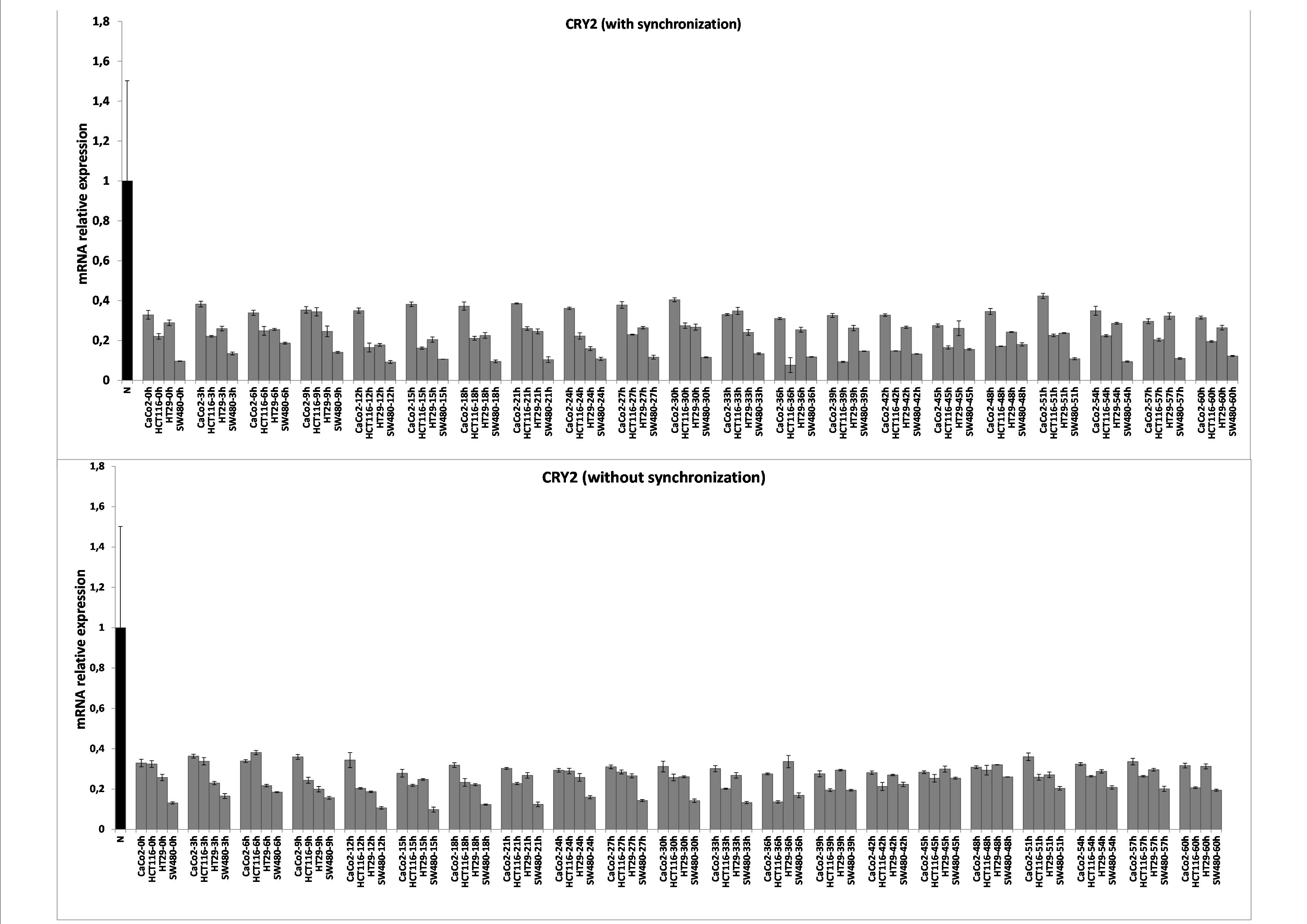

Supplement: Additional file 3: Figure S3. — x-y plots representing the time related mRNA expression profiles of CRY1 in CaCo2, HCT116, HT29 and SW480 cells with and without synchronization with serum shock and normalized to non-tumorous colorectal mucosa. Two biological replicates were each assayed in triplicate and results were expressed as mean ± standard deviation (SD). (JPG 568 kb) [file 12943_2016_492_MOESM3_ESM.jpg]

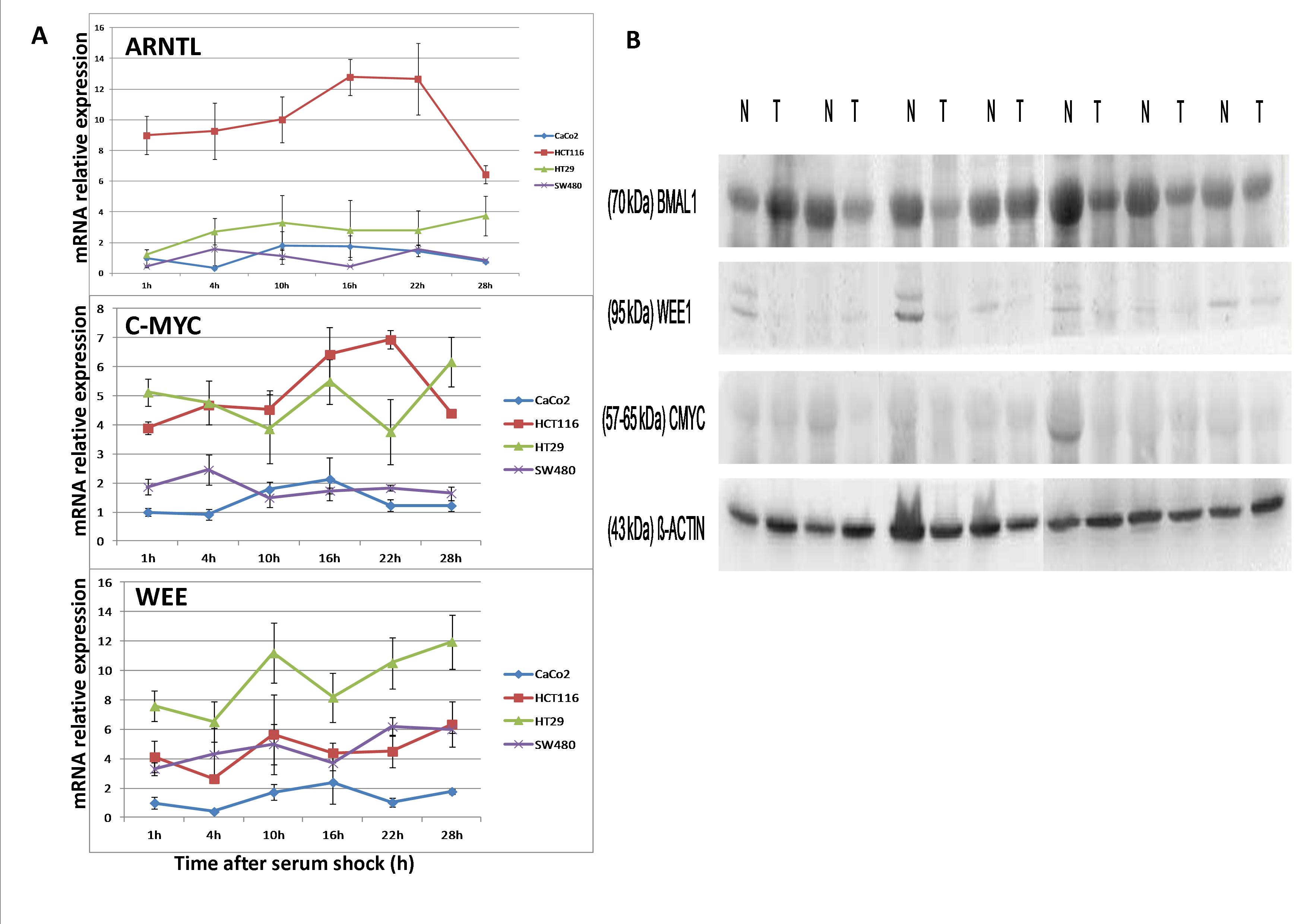

Supplement: Additional file 4: Figure S4. — A) x-y plots representing the time related mRNA expression profiles of ARNTL, WEE and c-MYC in CaCo2, HCT116, HT29 and SW480 cells synchronized after serum shock and harvested at the indicate time points. Two biological replicates were each assayed in triplicate and results were expressed as mean ± standard deviation (SD). B) ARNTL, WEE and c-MYC protein level evaluated by western blotting in a panel of matched specimens of tumour tissue and non-tumorous tissue. (JPG 473 kb) [file 12943_2016_492_MOESM4_ESM.jpg]
